# Evaluation of millets for physio-chemical and root morphological traits suitable for resilient farming and nutritional security in Eastern Himalayas

**DOI:** 10.3389/fnut.2023.1198023

**Published:** 2023-07-04

**Authors:** Jayanta Layek, Krishnappa Rangappa, Anup Das, Meraj A. Ansari, Sunita Choudhary, Namrata Rajbonshi, Sandip Patra, Amit Kumar, Vinay K. Mishra, Natesan Ravisankar, Sunil Kumar, Samarendra Hazarika, Sudip K. Dutta, Subhash Babu, M. Tahasildar, Nivedita Shettigar

**Affiliations:** ^1^ICAR Research Complex for NEH Region, Umiam, Meghalaya, India; ^2^ICAR Research Complex for Eastern Region, Patna, India; ^3^Project Coordination Unit, ICAR-Indian Institute of Farming Systems Research, Modipuram, Meerut, India; ^4^International Crops Research Institute for the Semi-Arid Tropics, Hyderabad, India; ^5^ICAR-Indian Institute of Farming Systems Research, Modipuram, Meerut, India; ^6^ICAR-Indian Agricultural Research Institute, New Delhi, India

**Keywords:** millets, physio-chemical traits, root architecture, nutritional security, destabilized soil ecosystem

## Abstract

**Introduction:**

Millets are nutritionally superior and climate-resilient short-duration crops and hold a prominent place in cropping sequences around the world. They have immense potential to grow in a marginal environment due to diverse adaptive mechanisms.

**Methods:**

An experiment was conducted in an organic production system in the North Eastern Himalayan foothills of India for 3 consecutive years by evaluating high-yielding varieties (HYVs) of different millets, viz., finger millet, foxtail millet, little millet, barnyard millet, proso millet, and browntop millet, along with local landraces of finger millets (*Sikkim-1* and *Sikkim-2; Nagaland-1* and *Nagaland-2*) to identify stable, high-yielding, and nutritionally superior genotypes suited for the region.

**Results:**

Among the various millets, finger millet, followed by little millet and foxtail millet, proved their superiority in terms of productivity (ranging between 1.16 and 1.43 Mg ha^−1^) compared to other millets. Among different varieties of finger millets, cv. VL Mandua 352 recorded the highest average grain yield (1.43 Mg ha^−1^) followed by local landraces, *Nagaland-2* (1.31 Mg ha^−1^) and *Sikkim-1* (1.25 Mg ha^−1^). Root traits such as total root length, root volume, average diameter of roots, and root surface area were significantly higher in finger millet landraces *Nagaland-1, Nagaland***-***2*, and *Sikkim-1* compared to the rest of the millet genotypes. The different millets were found to be rich sources of protein as recorded in foxtail millet cv. SiA 3088 (12.3%), proso millet cv. TNAU 145 (11.5%), and finger millet landraces, *Sikkim-1* and *Nagaland-2* (8.7% each). Finger millet landrace *Sikkim-2* recorded the highest omega-6 content (1.16%), followed by barnyard millet cv. VL 207 (1.09%). Barnyard millet cv. VL 207 recorded the highest polyunsaturated fatty acid (PUFA) content (1.23%), followed by foxtail millet cv. SiA 3088 (1.09%). The local finger millet landraces *Sikkim-1* and *Sikkim*-*2* recorded the highest levels of histidine (0.41%) and tryptophan (0.12%), respectively. *Sikkim-1* and *Nagaland-2* recorded the highest level of thiamine (0.32%) compared to the HYVs.

**Conclusion:**

These findings indicate that finger millet has great potential in the organic production system of the North Eastern Himalayan Region (NEHR) of India, and apart from HYVs like VL Mandua 352, local landraces, *viz., Nagaland-2 and Sikkim-1*, should also be promoted for ensuring food and nutritional security in this fragile ecosystem.

## 1. Introduction

The rising concern for food and nutritional security and environmental sustainability is creating tremendous pressure on mankind for judicious resource allocation and conservation (1). Soil degradation, malnourished human population, and poverty are some of the major concerns of the twenty first century (2). These global issues must be addressed by shifting toward lower energy and more resilient intensive cultivation practices (3, 4). For maintaining proper human health and physical wellbeing, the nutritional quality of food is the most important factor (5). Millets have many nutritional and health benefits (6) and are considered a superfood (7, 8). Most millets are extraordinarily superior to other cereals, such as rice and wheat. Millets are climate-resilient and sustainable crops that can be grown with a minimum amount of input (9, 10). Small and marginal farmers of the North Eastern Himalayan Region (NEHR), India, are facing a variety of problems that will intensify in the era of climate change (3, 11). Millets are small-seeded cereal crops belonging to the family *Poaceae* and are considered the world's sixth most important cereal grain crop, feeding more than one-third of the world's population (12, 13). They are pre-green-revolution crops cultivated traditionally by many generations (9). Millets are considered to be ancient grains of mankind that can grow from coastal regions of Andhra Pradesh to moderately high altitudes in the Himalayan Region, namely, the states of Uttarakhand and NEHR of India, which is indicative of their wide capacity for adaptation (14). Cultivated and consumed in over 50 countries around the globe including India, millet is central to the cultural ethos of indigenous communities in the Eastern Himalayas and other parts of India (15). Grown as dual-purpose crops (food and fodder), millets occupy an integral part of subsistence agriculture by providing food and livelihood security to millions of inhabitants including small and marginal farmers of remote rain-fed/hilly areas of the NEHR, India (16). Millets are short-duration, non-exhaustive crops that can be grown with minimum input requirements (17, 18) and thus fit well in organic farming (18). Generally grown as rain-fed crops, they require very low amounts of water to complete their life cycle (19, 20). India is the largest producer of millets in the world with a share of 41%, followed by African countries like Niger (11%) and Nigeria (7%) (21). They contain a high amount of carbohydrates (60–70%), dietary fibers (10–12%), protein (6–9%), fat (1.5–5%), and a considerable amount of minerals (2–4%) (22). They stand out from other cereals because of the high calcium and polyphenol content in the grains (23). Millets are great for boosting the nervous system (8, 17). Consumption of millets controls blood sugar levels and cholesterol and enhances the immune system (10). They can be consumed by people having type 2 diabetes and are good for heart ailments.

At the moment, the fragile and marginal ecosystem in the NEHR of India is one of the most significant factors that act as a barrier to optimum crop production (18). Among the different millets, minor millets such as finger millet, followed by foxtail millet and barnyard millet, are the major and most versatile millets in the NEHR of India (18). Owing to their high nutritional content (18, 22), good yield potential, availability of seed, storage, and utilization technology, millets could significantly contribute to the food and nutritional security of the region's inhabitants (24). In northeast India, millets have been an integral part of the farming system for a long time, and local cultivars are grown in the *Jhum* fields along with other crops such as paddy (25). They are not commonly grown as commercial crops but are mainly cultivated by tribal farmers as a part of subsistence farming (14, 26). Small millets especially foxtail millet, finger millet, and barnyard millet are confined to the NEHR of India in Nagaland (Phek, Tuensang, and Kiphire districts) (27), Meghalaya (Khasi Hills and West Garo hills), Manipur (Churachandpur and Senapati districts), and hilly areas of Arunachal Pradesh, Sikkim (28) and Mizoram (Mara tribe) (14). In Manipur, ethnic tribes such as the Thadou Kuki and Paite tribes cultivate millets in *Jhum* agriculture and traditionally make cake from millets as an offering to their ancestors. Raishan (*Digitaria cruciata* var. *esculenta* Bor) is an indigenous cold-tolerant millet crop, endemic to the Khasi hills of Meghalaya, that is cultivated for both food and fodder (24, 29). “Tsiinyi” or millet festival celebrated by the Angami Naga tribe of Nagaland signifies the importance of millets in their traditions. Tribal people from some areas of Sikkim prepare “*kodoko Jaanr*” from seeds of finger millet (30). The cultural utility of minor millets such as proso millets is high in Arunachal Pradesh. They are cultivated in *jhum* fields and provide various indigenous food items for use in traditional ceremonies and occasions. Brown top millet and little millet are concentrated in specific parts of the NEHR and are grown in hilly terrain (31). The elderly tribal population of the NEHR grew up having them as “staple food.” Although millets were known as the poor man's food, increased consumer awareness and the high market price of millets in recent times have opened a new avenue to grow them as cash crops even in hills (16). Low-productive cereal and mixed agricultural cropping patterns are prevalent in the NEHR hills, particularly on sloppy and *jhum* terrain of India (11). Low-input, resource-efficient crops, such as suitable millets (nutri-cereals), are gaining popularity as prospective solutions for assuring food and nutritional security. Most of the farmers in the region do not use any synthetic fertilizer or pesticides, and hence, they are organic by default (5, 32, 33). The NEHR generates 2.55 million tons of agricultural biomass and has 2.98 million bovines, encouraging organic crop production through recycling for valuable nutrient management inputs (5). In the past few decades, there has been a rising emphasis on using organic production systems to increase soil quality, crop productivity, and nutritional security vis-a-vis maintaining environmental quality.

Identification of suitable and resource-efficient millets with better adaptive mechanisms (better root architecture with enriched biochemical properties) for the marginal and destabilized ecosystem is of the utmost importance to integrate suitable millet into the cropping systems. Small millets have a high production potential under ideal conditions, and millets have a diverse set of adaptation mechanisms that allow them to grow and survive in environments that are relatively marginal and destabilized. The relevance of root design for water and nutrient uptake has been extensively documented in both monocots and dicots, and it could be employed effectively for trait-specific genetic enhancement of roots. Millets have fibrous root systems in which distinct root types contribute toward improving resource use efficiency (water, nutrients etc.) (34). Significantly large variations in root properties were identified for minor millets including local germplasms of finger millets from Himalayan foothills, indicating a potential capacity to incorporate minor millets on a large scale in this fragile ecosystem. There is also a need to identify nutritionally superior millets suitable for these areas.

Through proper awareness programs and focused research, millets can be popularized as a potential cash crop with organic certification in this ecosystem of the NEHR of India and similar other agroecological regions of the world. Both the nutritionally rich local landraces and high yielding varieties (HYVs) of millets must be selected, conserved, and promoted for cultivation by farmers. Furthermore, this highly nutritious grain crop is mostly limited to particular patches and should be promoted to the majority of the population residing in the region. Keeping these points in view, the study “Evaluation of millets for physio-chemical and root morphological traits suitable for resilient farming and nutritional security in Eastern Himalayas” was conducted.

## 2. Materials and methods

### 2.1. Experimental site and treatment details

An experiment was undertaken with different varieties/landraces of finger millet, foxtail millet, little millet, brown top millet, proso millet, and barnyard millet for evaluating their suitability under the organic production system in the NEHR of India (Table 1). Altogether, 13 different germplasms of millets were evaluated for 3 consecutive years in the *kharif* seasons of 2018–2020 in organic upland terraces of the Agronomy field, ICAR Research Complex for the NEHR of Umiam, Meghalaya (25°30'N latitude and 91°51'E longitude) situated at 980 m ASL. Apart from HYVs of different minor millets, the local landraces of finger millets from the NEHR of India (*viz., Nagaland-1, Nagaland-2, Sikkim-1*, and *Sikkim-2*) were also collected and evaluated. The temperature in this region is moderate throughout most of the year except for the few months of winter. The maximum temperature ranges from 26 to 29 °C from March to October. In the winter, the minimum temperature rarely goes below 5°C. The region receives a good amount of rainfall (~ 2,400 mm annually), but the majority of it occurs from April to October (Supplementary Figure 1). The maximum relative humidity of the region generally ranges above 80%, while the minimum relative humidity rarely goes below 50%, with mean annual evaporation is approximately 850 mm. A collection of practices used for growing millets under organic conditions in this study's experiment is given in Table 2.

**Table 1:** Millets used in the experiment with their common English name and characteristics.

**Millet**	**Common name**	**Scientific name**	**Germplasms**	**Duration (days)**	**Av. yield (Qha^−1^)**	**Agronomic characteristics**
Foxtail millet	Kangni/Kakun	*Setaria italica*	SiA 3088	70–75	20–25	Short duration, non-lodging, suitable for double cropping
Little millet	Kutki/Shavan	*Panicum sumatrense*	OLM 203	105–110	10–11	Blast and grain smut resistant
Browntop millet	Korale millet	*Urochloa ramose*	Local	95–100	14–18	Nutritionally superior, hardy crop
Barnyard millet	Sanwa	*Echinochloa frumentacea*	VL 207	85–100	16–20	High nutritional value and can withstand biotic and abiotic stresses
Proso millet	Chena/Barri	*Panicum miliaceum*	TNAU 145	70–72	18–20	Superior grain quality for value addition, suitable for limited crop growth period areas because of its short duration
Finger millet	Ragi/Mandua/Nachani	*Eleusine coracana*	VL Mandua 324	105–135	20–25	Blast resistant
			VL Mandua 352	90–100	25–30	High yielding, moderately resistant to blast, and is also used for contingent crop planning
			VL Mandua 172	80–95	13–17	Medium duration
			VL Mandua 347	< 100 days	20–22	Short duration and moderately resistant to blast disease Suitable for higher hills (or areas where crop growth period is limited) because of its short duration
			*Sikkim-1*	85–95	15–20	Stress tolerant, faster growth, suitable for hilly areas
			*Sikkim-2*	85–100	12–17	Stress tolerant, faster growth, suitable for hilly areas
			*Nagaland−1*	90–100	13–16	Pest and disease resistant
			*Nagaland−2*	90–100	20–25	High yielding

**Table 2:** Package of practices of millets grown under organic conditions in North Eastern Hill Region (NEHR), India.

**Varieties**	**Finger millet**	**HYVs like VL Mandua 324, 352, 172, 347, HR 374, local landraces of Sikkim (*Sikkim-1,2*) and Nagaland (*Nagaland-1, 2*)**
	Foxtail millet	SiA 3088
	Proso millet	TNAU 145
	Little millet	OLM 203
	Barnyard millet	VL 207
	Browntop millet	Local
Land preparation	Field was prepared by two cross plowings followed by two harrowing and planking. Well-decomposed farm yard manure (FYM) @ 5 t/ha was added and mixed in the soil before sowing.	
Seed rate	8–10 kg seeds per hectare.	
Sowing time	The crop was sown from mid-June to the first week of July.	
Sowing method	Line sowing was followed.	
Spacing	A row-to-row spacing of 25–30 cm and plant-to-plant spacing of 8–10 cm was maintained.	
Nutrient management	7.0–8.0 t/ha FYM, or 5.0 t/ha FYM + 1.0 t/ha vermicompost, 400–500 kg lime, and 150 kg/ha rock phosphate were mixed and applied in furrows before seed sowing.	
Water management	No irrigation was applied, as NEHR gets plenty of rainfall from May to October (more than 1,500 mm).	
Intercultural operations	Thinning and gap filling	Thinning was performed after 2 to 3 weeks of germination to maintain the desired spacing. Gap filling was practiced in case of the death of seedlings.
	Weeding	First-hand weeding was carried out at 25–30 DAS and second weeding using a wheel hoe at 50 DAS.
Insect pest and disease management	Spraying of neem oil 0.03% @ 3 ml/liter of water twice at 10-day intervals for control of pink stem borer and Bihar hairy caterpillar. Disease-free seeds were selected, and proper sanitation practices were followed. Bio-control agents such as *Pseudomonas* spp. and *Trichoderma* spp. were used for seed and soil treatment.	
Harvesting	The crop matured in approximately 70–120 days depending on altitude and variety, and was ready for harvest by the first week of October.	
Yield	Millets have a grain yield potential of 1.30–1.50 t/ha under organic cultivation	

### 2.2. Physiological observations

#### 2.2.1. Leaf chlorophyll analysis

Chlorophyll index as a reflection of chlorophyll density per unit area was determined using the acetone extraction method (80:20 acetone and water mixture) to reflect different components of leaf chlorophyll in terms of its sub-components, *viz.*, chlorophyll a, b, total chlorophyll, and carotenoids content (35). A known quantity of fresh leaf sample (0.5 g) was grounded and homogenized with 15 ml of 80:20 acetone: water mixture solution. The leaf extract was then filtered through the Whatman No. 42 filter paper after which the volume was made up to 25 ml with the same solvent mixture solution. Later, the required aliquot of the final extract was taken in a cuvette, and absorbance was measured at 648, 652, and 663 nm using a Spectronic-20 UV spectrophotometer to assess chlorophyll “a,” chlorophyll “b,” and total chlorophyll of each sample in mg g^−1^ of fresh tissue. The required calculations were performed using the following formulae (35, 36). Similarly, leaf carotenoid was also calculated through the same extraction protocol.


(1) $$\begin{array}{l} Chla\;(mg\;g^{-1})\;=\;(12.72\times A_{663}-\;2.58\;\times A_{645})\;\times \;(V/W)\;\\ \;\times (1/1000) \end{array}$$

(2) $$\begin{array}{l} Chlb\;(mg\;g^{-1})\;=\;(22.87\;\times A_{645}-4.67\;\times A_{663})\;\times \;(V/W)\;\\ \;\times (1/1000) \end{array}$$

(3) $$\begin{array}{l} Chla+b\;(mg\;g^{-1})\;=\;(8.05\;\times A_{\text{ 663 +}}\;20.29\;\times A_{645})\;\times \;(V/W)\;\\ \;\times (1/1000) \end{array}$$

(4) $$\begin{array}{l} Carotenoids\;=\;(A_{480}\;+\;0.114\;\times \;A_{663}-\;0.638\;\times \;A_{645})\;\times \\ \;(V/W)\;\times \;(1/1000) \end{array}$$

where “V” refers to the total volume of the extract, “W” refers to the weight of tissue taken for pigment measurements, and A_663_, A_645_, and A_480_ are the optical absorbance values recorded by the Spectronic-20 UV spectrophotometer at 663, 645, and 480 nm, respectively.

#### 2.2.2. Gas exchange measurements

Photosynthetic parameters, *viz.*, photosynthetic rate (A), stomatal conductance (GH_2_O), transpiration rate (E), leaf temperature (°C), and intracellular CO_2_ (Ci) were recorded at the flowering stage using a portable infrared gas analyzer (IRGA-GFS Walz-3000 Model, Germany) in the fully expanded (third leaf from top) leaf of randomly selected representative plants. The IRGA mainly consists of a main console that comprises separate detectors for two different apparent hetero-atomic molecules CO_2_ and H_2_O involved during photosynthesis, an internal air supply unit, a leaf chamber to clamp the leaves, and the necessary software for the computation of gas exchange parameters. It is equipped to measure the light intensity in the PAR range using a point quantum sensor, relative humidity using a thermocouple, and temperature of the air using a thermostat. Butyl rubber tubing is used to carry air from the leaf chamber into the IRGA. Instantaneous water use efficiency was calculated using the formula Pn/E (net photosynthetic rate over leaf transpiration) (37, 38).

#### 2.2.3. Determination of stomatal attributes

As leaves of millets have the amphistomatic type of stomatal distribution in both surfaces (39), stomatal counting was carried out on both the abaxial and adaxial surfaces by smearing with nail polish followed by shade drying (40). The smeared leaf was cut into 2.0–2.5 cm^2^ dimensions, and the layer of nail polish impression was gently removed. With the help of forceps, the same was placed on microscopic slides with a few drops of water and covered with a glass cover slip. Stomatal numbers on every sample surface were counted in three different microscopic fields of 10X and 40X magnifying lenses of a compound microscope (BX-50F, Olympus, Japan). Other related observations such as stomatal length, breadth, number of guard cells, and stomata in the particular microscopic field were recorded using user-friendly software.


(5) $$\begin{array}{l} Stomatal\;frequency\;=\;Numbers\;of\;stomata\;per\;unit\;area\\ \;(No.\;mm^{-1}) \end{array}$$

(6) $$\begin{array}{l} Stomatal\;index=\\ \frac{Numbers\;of\;stomata}{Number\;of\;stomata\;+\;Number\;of\;epidermal\;cell}\times 100 \end{array}$$

#### 2.2.4. Root architecture analysis

Plants from different treatments were carefully uprooted without disturbing the intact root system, and the same roots were washed with a smooth flush of water (2, 38). After shade drying for 1 or 2 h, intact turgid roots (full root system) were evenly spread on a transparent fiber tray (30 cm × 20 cm) without overlapping, and the same was scanned for a two-dimensional root image at a resolution of 200 dpi (dots per inch) using an Epson v 700 perfection scanner (Regent Instrument Canada Inc., Quebec, Canada). The resulting images were acquired and processed using the WinRHIZO professional software program. Every root system image was analyzed for total root length (TRL), root surface area (RSA), root volume (RV), average diameter (RD), and number of tips (N Tips). Both Regent's non-statistical method and Tennant's statistical method were chosen to perform root morphology measurements (41) in WinRHIZO. To avoid a high scanning density, the large root sample was subdivided into smaller sub-samples before scanning (2, 42, 43).

#### 2.2.5. Measurement of shoot traits

The fully expanded and matured leaves (fourth leaf from the top) were used for the measurement of leaf thickness (LT). LT was measured using an absolute digital vernier caliper (Mitutoyo Corp., Japan) at the broadest part of the leaf excluding major veins with an accuracy of ±0.01 mm and expressed in μm. The LT was measured as direct reading with a gentle pressing of the caliper to avoid overestimation and any injury to the intact leaf (44). For recording the shoot and root dry weight, the fresh and air-dried root and shoot samples were oven dried at 72°C for 48 h or until reaching constant weight and expressed in terms of g/plant. The root-to-shoot ratio was calculated by dividing root dry weight (RDW) by shoot dry weight (SDW). Total dry matter (TDM) was derived by adding SDW and RDW and expressed on a single-plant basis.

### 2.3. Yield attributes and yield

The grain, as well as straw yields of millets from the experimental fields, was measured from net plot areas. Net grain and straw yields were reported at a moisture content of 13%. The unit grain weight was obtained for 1,000 grains as test weight (g). The harvest index (HI) and production efficiency were calculated by the following formula:


(7) $$\begin{array}{l} HI=\frac{Economic\;yield}{Biological\;yield}\;\times 100 \end{array}$$

(8) $$\begin{array}{l} Economic\;yield\;=\;Grain\;yield\;in\;Mg\;ha^{-1} \end{array}$$

(9) $$\begin{array}{l} Biological\;yield\;=\;Total\;plant\;biomass\;(seed\;yield\;+\\ \;stover\;yield\;+\;root\;biomass)\;in\;Mg\;ha^{-1} \end{array}$$

### 2.4. Grain nutritional quality analysis

Grain nutritional traits were estimated based on near-infrared spectroscopy (NIRS) with validated calibration models. Grains of each genotype were threshed, cleaned, and ground to flour of < 1 mm particle size, using a CM290 Cemotech™ laboratory grinder (FOSS, Hillerød, Denmark). The flour samples were then stored in 50-mL conical polypropylene falcon tubes at 4^o^C until scanning with NIR instruments. Prior to scanning, the samples were dried at 50^o^C for 16 h and cooled to room temperature. The samples were then scanned using a benchtop NIR spectrometer DS2500 flour analyzer from FOSS (FOSS-DS2500; FOSS Electric A/S, Hillerød, Denmark). For obtaining the spectral sample signature from the FOSS-DS2500, each flour sample was transferred to the standard circular ring cup (inside diameter~6 cm, FOSS sample cup) and scanned three times at room temperature (~26^o^C). The sample was mixed before each scan. The NIR spectral absorbance, with a range of 400–2498 nm, was recorded as the logarithm of reciprocal reflectance (1/R) with 2 nm intervals, using SCANISI and predicted using Solo Mosaic analytical software (v4.4, InfraSoft International LLC, PA, USA) and calibration models for various traits [(45); https://fern-lab.github.io/].

### 2.5. Statistical analysis

All the data obtained from the study were statistically analyzed using the ANOVA and the “F test” for testing their significance ((46), 35). Standard error of means (SEm±), as well as least significant difference (LSD), was calculated at a 5% level of significance for all the parameters studied to know the differences between treatment means. The R program was used to perform the principal component analysis (PCA) (47).

## 3. Results

### 3.1. Physiological parameters

**Figure 1:**
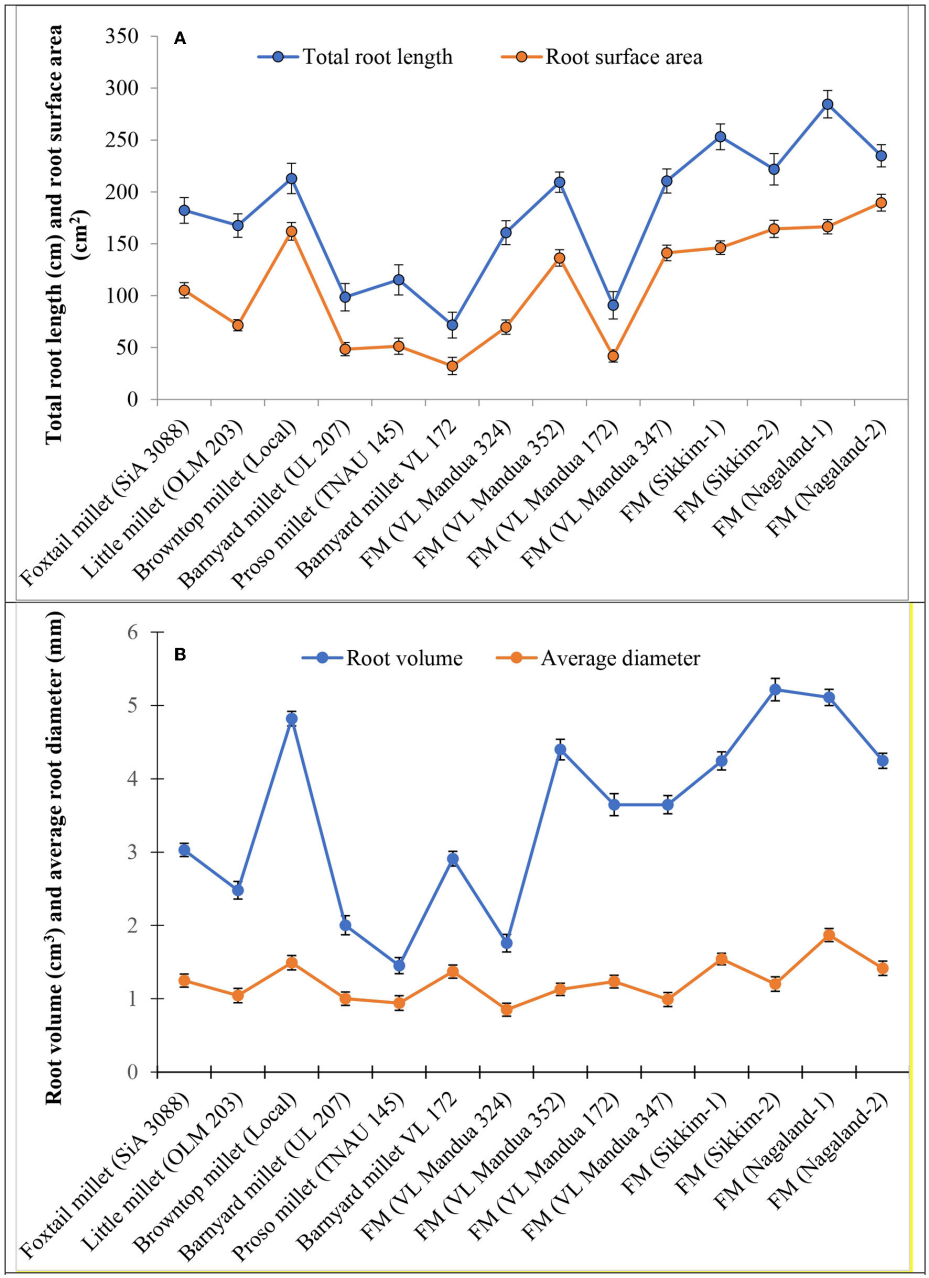
Root morphological attributes of millet genotypes. **(A)** Total root length and root surface area. **(B)** Root volume and average of diameter. Vertical bars (both way) represent standard error (*p* = 0.05).

**Figure 2:**
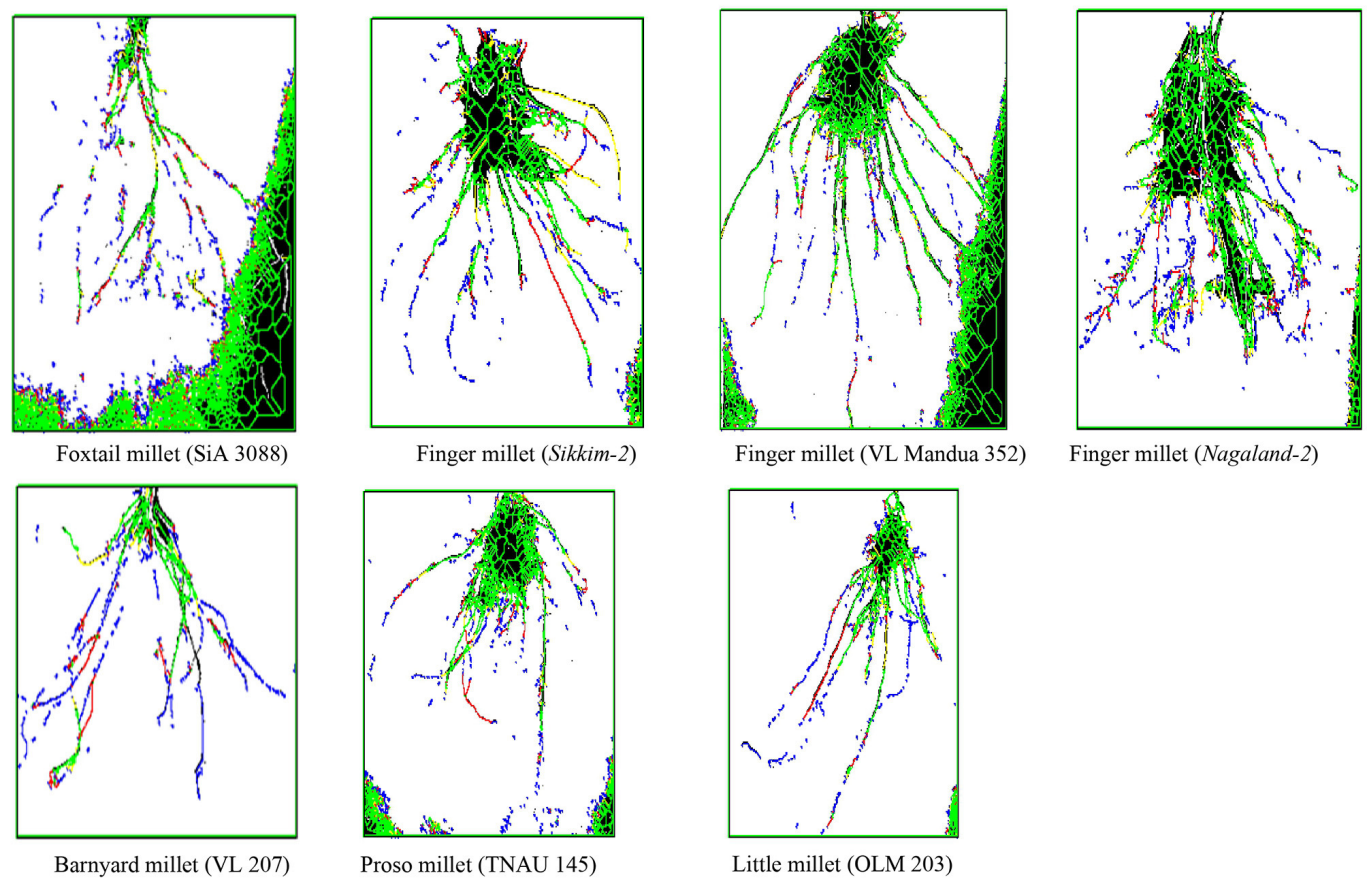
Images from root architecture analysis of millets.

**Figure 3:**
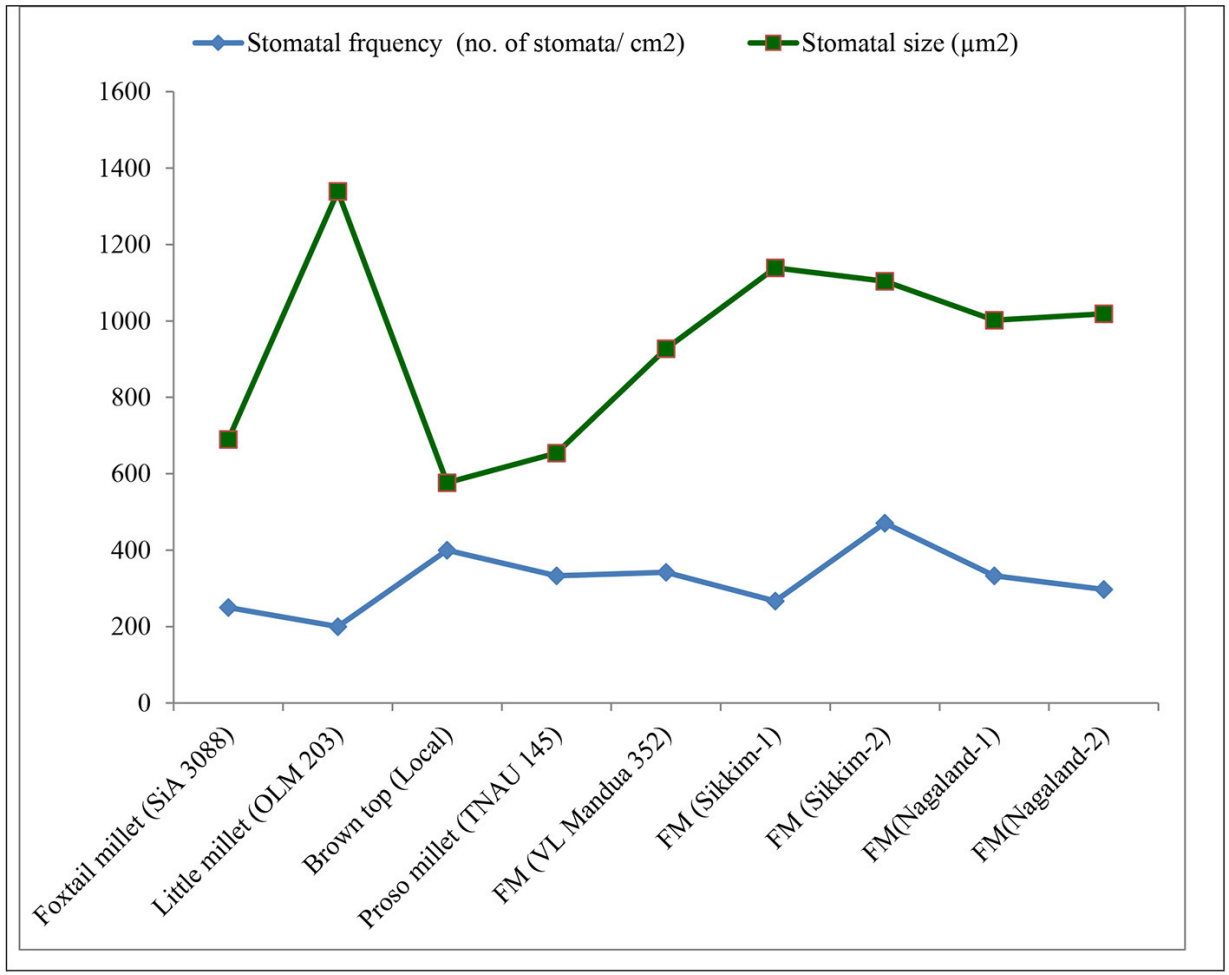
Stomatal attributes of millets grown under organic conditions.

Among the root morphological attributes, the finger millet landrace, *Nagaland-1*, recorded the highest average root diameter and total root length with values of 1.87 mm and 284.53 cm respectively, whereas the landrace, *Nagaland-2*, recorded the highest root surface area (189.60 cm^3^) followed by *Nagaland-1* (166.42 cm^2^) (Figure 1). The finger millet landrace, *Sikkim-2*, obtained the highest root volume (5.22 cm^3^), which was statistically at par with the finger millet landrace, *Nagaland-1* (5.11 cm^3^) (Figures 1, 2). The highest stomatal frequency was recorded in finger millet landrace, *Sikkim-2* (471 cm^−2^), and little millet cv. OLM 203 observed the highest stomatal size with a value of 1338.7 μm^2^ followed by local landrace, *Sikkim-1* (1138.7 μm^2^) and *Sikkim-2* (1104.1 μm^2^) (Figure 3). Among the different millets, foxtail millet cv. SiA 3088 recorded the highest Chl*a* content (3.69 mg g^−1^ FW) followed by finger millet cv. VL Mandua 352 and local landrace, *Sikkim-1* (3.55 mg g^−1^ FW each) (Table 3). Similarly, the highest Chl*b* (2.10 mg g^−1^ FW) was recorded for foxtail millet cv. SiA 3088, which was followed by finger millet landrace, *Sikkim-2* (2.08 mg g^−1^ FW) and cv. VL Mandua 352 (2.06 mg g^−1^ FW). However, the highest ratio of Chl*a/b* was recorded for foxtail millet cv. SiA 3088 (1.76) followed by local landraces of finger millet *Nagaland-2* (1.78 mg g^−1^ FW) and *Sikkim-1* (1.74 mg g^−1^ FW). Significantly, the highest carotenoids were recorded for foxtail millet cv. SiA 3088 (149.10 mg g^−1^ FW) followed by little millet cv. OLM 203 (136.70 mg g^−1^ FW) and finger millet landrace, *Sikkim-1* (125.10 mg g^−1^ FW). The highest stomatal conductance-GH_2_O (286.10 mmol m^−2^ s^−1^) was recorded under the local landrace of browntop millet (Table 3 and Figure 3). Among the different germplasms of finger millets evaluated, landrace *Sikkim-1* recorded the highest leaf temperature (41.1°C) followed by *Nagaland-2* (41.0°C) (Table 3).

**Table 3:** Leaf pigmentation and photosynthetic measurements of different millets grown under organic farming practices (after 60 DAS).

**Germplasms**	**Varieties/landraces**	**Chl*a* (mg g^−1^ FW)**	**Chl*b* (mg g^−1^ FW)**	**Carotenoids (μg/gFW)**	**Chl a/b**	**Leaf temperature (°C)**	**Stomatal conductance-GH_2_O (mmol m^−2^ s^−1^)**
Foxtail millet	SiA 3088	3.69	2.10	149.10	1.78	39.00	147.00
Little millet	OLM 203	3.33	2.01	136.70	1.66	39.90	236.40
Browntop millet	Local	3.21	1.86	121.80	1.73	38.40	286.10
Barnyard millet	VL 207	3.47	1.73	117.30	2.01	39.20	234.70
Proso millet	TNAU 145	3.08	2.05	96.50	1.50	39.10	253.60
Finger millet	VL Mandua 324	3.32	1.96	112.70	1.69	38.50	162.50
	VL Mandua 352	3.55	2.06	116.50	1.72	38.60	280.30
	VL Mandua 172	3.38	1.98	102.60	1.71	39.40	217.10
	VL Mandua 347	3.26	1.94	91.90	1.68	40.40	159.70
	*Sikkim-1*	3.55	2.04	125.10	1.74	41.10	168.20
	*Sikkim-2*	3.45	2.08	105.70	1.66	40.40	239.40
	*Nagaland-1*	3.17	1.84	107.90	1.72	40.60	192.10
	*Nagaland-2*	3.52	1.98	98.00	1.76	41.00	238.70
	SEm±	0.04	0.05	2.59	0.04	SEm±	0.80
	CD (P=0.05)	0.12	0.14	7.57	0.13	CD (*P* = 0.05)	2.34

### 3.2. Yield

Finger millet recorded the highest average grain yield (0.73−1.43 Mg ha^−1^) followed by foxtail millet (1.17 Mg ha^−1^) and little millet (1.16 Mg ha^−1^) (Table 4). The lowest yield was observed for proso millet (0.29 Mg ha^−1^). Among the different finger millet germplasms, the highest average grain yield was recorded for cv. VL Mandua 352 (1.16 Mg ha^−1^) followed by local landraces, *Nagaland-2* (1.31 Mg ha^−1^) and *Sikkim-1* (1.25 Mg ha^−1^). The highest HI was recorded for finger millet cv. VL Mandua 352 (27.3%) and cv. VL Mandua 172 (27.2%) (Table 3). Among the local landraces of finger millet evaluated over 3 years, the highest HI was recorded for *Sikkim-1* (22.6%). Significantly, the highest test weight (weight of 1,000 seed weight) was reported in barnyard millet cv. VL 207 (3.61 g) followed by finger millet cv. VL Mandua 172 (3.20 g).

**Table 4:** Yield of different millets under organic production system (average of 3 years data).

**Germplasms**	**Varieties/landraces**	**Grain yield (Mg ha^−1^)**	**Harvest index (%)**	**Test weight (g 1,000 seed^−1^)**
Foxtail millet	SiA 3088	1.17	25.0	3.14
Little millet	OLM 203	1.16	24.2	3.09
Browntop millet	Local	1.02	17.5	2.80
Barnyard millet	VL 207	1.09	26.5	3.61
Proso millet	TNAU 145	0.29	18.9	3.17
Finger millet	VL Mandua 324	1.16	22.8	2.84
	VL Mandua 352	1.43	27.3	2.99
	VL Mandua 172	1.21	27.2	3.20
	VL Mandua 347	1.22	26.7	2.99
	*Sikkim-1*	1.25	22.6	2.93
	*Sikkim-2*	0.73	20.2	2.79
	*Nagaland-1*	0.97	20.3	2.81
	*Nagaland-2*	1.31	19.6	2.69
SEm±		0.050	0.55	0.07
CD (*P* = 0.05)		0.13	1.60	0.20

### 3.3. Nutritional quality

**Figure 4:**
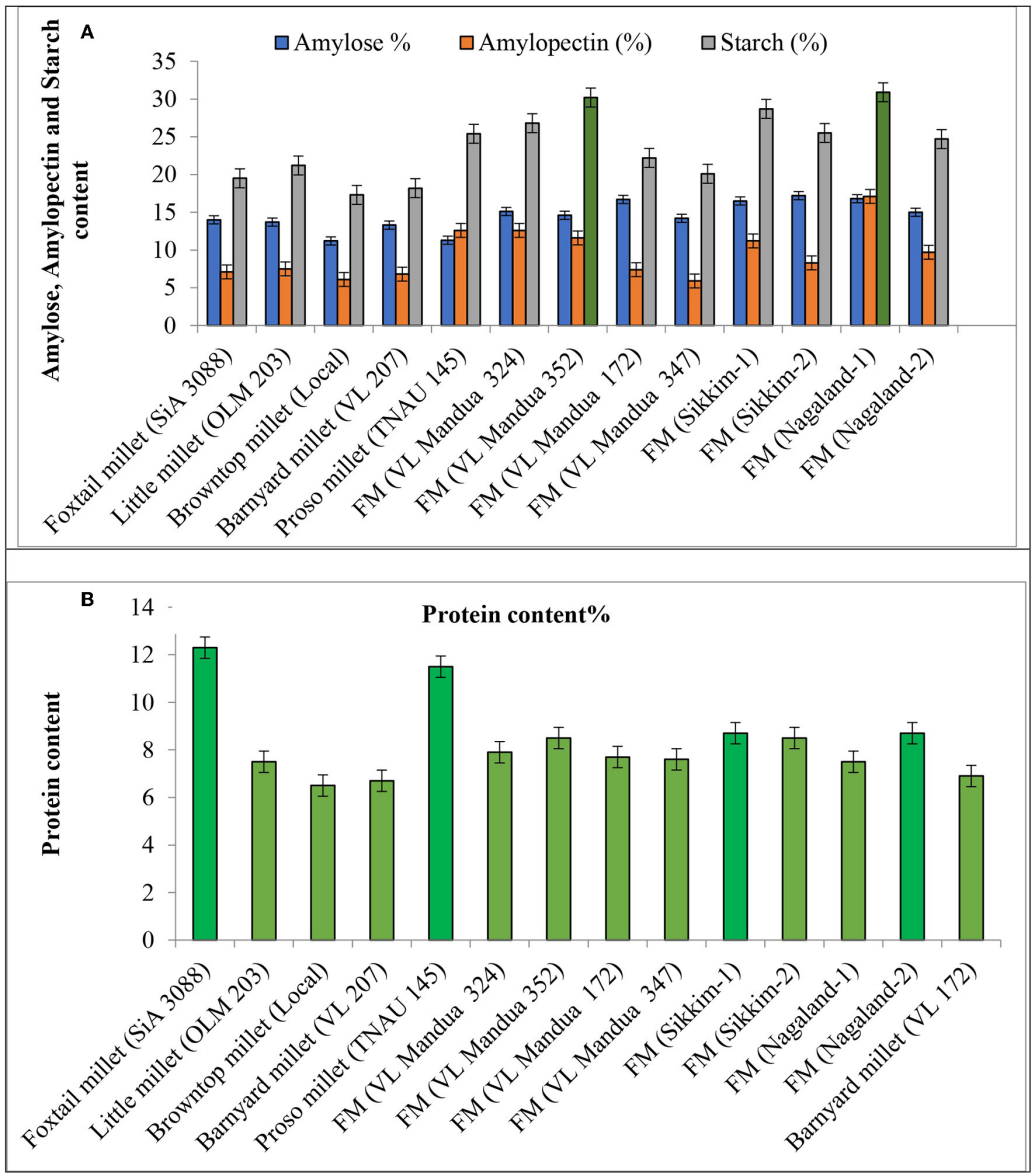
Effect of different lines of millets on quality parameters. **(A)** Amylose amylopectin and starch content. **(B)** Protein content. Vertical bars (both way) represents standard error (*p* = 0.05).

The experiment of different types of millets including different lines of finger millet over the years showed that there were significant variations in nutritional properties in grains such as protein content, amylose, amylopectin and starch content, and profile of amino acids and fatty acids (Table 5 and Figure 4). Foxtail millet cv. SiA 3088 recorded the highest protein content (12.30%) followed by proso millet cv. TNAU 145 (11.50%) (Figure 4). The local landraces of finger millet, *Sikkim-1* recorded the highest amylose content (17.20%) followed by *Nagaland-2* (16.80 %) compared to HYVs. Similarly, finger millet landrace, *Nagaland-1*, obtained the highest amylopectin content (17.10%) among all germplasms of millets. The starch content was found to be highest in finger millet landrace, *Nagaland-1*, with a value of 30.90 % followed by cv. VL Mandua 352 (30.20%) as shown in Figure 4. The finger millet landraces were also found to be superior in different nutritional properties content as shown in Table 4. For most of the nutritional parameters, local landraces of finger millet, *Sikkim-1 and Sikkim-2* and *Nagaland-1 and Nagaland-2*, outperformed the HYVs (Table 4 and Figure 4). The finger millet landrace, *Sikkim-2*, recorded the highest omega-6 content (1.16%) followed by barnyard millet cv. VL 207 (1.09%). cv. VL 207 also recorded the highest polyunsaturated fatty acid (PUFA) content (1.23%) followed by foxtail millet cv. SiA 3088 (1.09%). However, among the different germplasms of finger millets evaluated, local landraces, *viz., Sikkim-2* (1.01%) followed by *Nagaland-2* (0.79%) recorded significantly higher PUFAs against the best HYVs such as VL Mandua 324 (0.63%). Saturated fat concentration ranged from 0.30% in finger millet landrace, *Sikkim-2*, to 1.3% in foxtail millet cv. SiA 3088. The different germplasms of millets were also found to contain significant amounts of essential amino acids as shown in Table 4. For sulfur-containing amino acids (methionine and cysteine), the highest values of methionine were observed in finger millet cv. VL Mandua 172 and 347 (0.19%). The finger millet landraces, *Nagaland-2* and *Nagaland*-*1*, also recorded significantly higher values of methionine (0.19 and 0.17%, respectively) compared to other millets.

**Table 5:** Profiling of fatty acids and amino acids in different millets under an organic production system.

**Germplasms**	**Varieties/ landraces**	**Omega 6 (%)**	**PUFA (%)**	**Saturated fat (%)**	**Glutamic acid (%)**	**Cysteine (%)**	**Histidine (%)**	**Thiamine (%)**	**Methionine (%)**	**Tryptophan (%)**
Foxtail millet	SiA 3088	1.04	1.09	0.13	2.88	0.13	0.35	0.29	0.13	0.10
Little millet	OLM 203	0.79	0.72	0.08	3.01	0.16	0.36	0.23	0.14	0.07
Browntop millet	Local	0.55	0.61	0.07	3.62	0.23	0.33	0.26	0.14	0.03
Barnyard millet	VL 207	1.09	1.23	0.04	2.17	0.12	0.17	0.17	0.12	0.11
Proso millet	TNAU 145	0.50	0.55	0.07	1.53	0.08	0.34	0.32	0.18	0.11
Finger millet	VL Mandua 324	0.55	0.63	0.11	3.44	0.21	0.34	0.27	0.14	0.05
	VL Mandua 352	0.49	0.57	0.07	3.52	0.22	0.30	0.30	0.18	0.06
	VL Mandua 172	0.54	0.53	0.08	3.52	0.22	0.31	0.30	0.19	0.06
	VL Mandua 347	0.53	0.56	0.06	2.44	0.13	0.32	0.31	0.19	0.07
	*Sikkim-1*	0.68	0.74	0.08	3.08	0.21	0.41	0.32	0.13	0.11
	*Sikkim-2*	1.16	1.01	0.09	2.97	0.22	0.36	0.27	0.11	0.09
	*Nagaland-1*	0.61	0.70	0.09	2.19	0.11	0.34	0.29	0.17	0.11
	*Nagaland-2*	0.89	0.79	0.03	3.19	0.23	0.23	0.32	0.19	0.12
SEm±		0.02	0.02	0.00	0.07	0.00	0.01	0.01	0.00	0.00
CD (P=0.05)		0.06	0.05	0.00	0.21	0.01	0.02	0.02	0.01	0.01

PUFA, Polyunsaturated fatty acids.

While the landraces of browntop millet (local) and finger millet, *Nagaland-2*, recorded the highest values for cysteine (0.23%), the highest amount of glutamic acid content was found for local browntop millet (3.62%). Finger millet landraces, *viz., Nagaland*−*2* and *Sikkim-1*, also recorded higher values of glutamic acid (3.19 and 3.08%, respectively) compared to most of the millets evaluated. The histidine content was recorded to be maximum in finger millet landrace, *Sikkim-1* (0.41%) followed by *Sikkim-2* and little millet cv. OLM 203 (0.36%). Both the landraces of finger millets, *Sikkim-1* and *Nagaland-2*, recorded the highest values for thiamine (0.32% each). The finger millet landrace, *Nagaland-2*, contained the highest amount of tryptophan (0.12%) followed by *Nagaland-1, Sikkim-1*, barnyard millet variety, VL 207, and proso millet cv. TNAU 145 (each with a value of 0.11%).

### 3.4. Principal component analysis

**Figure 5:**
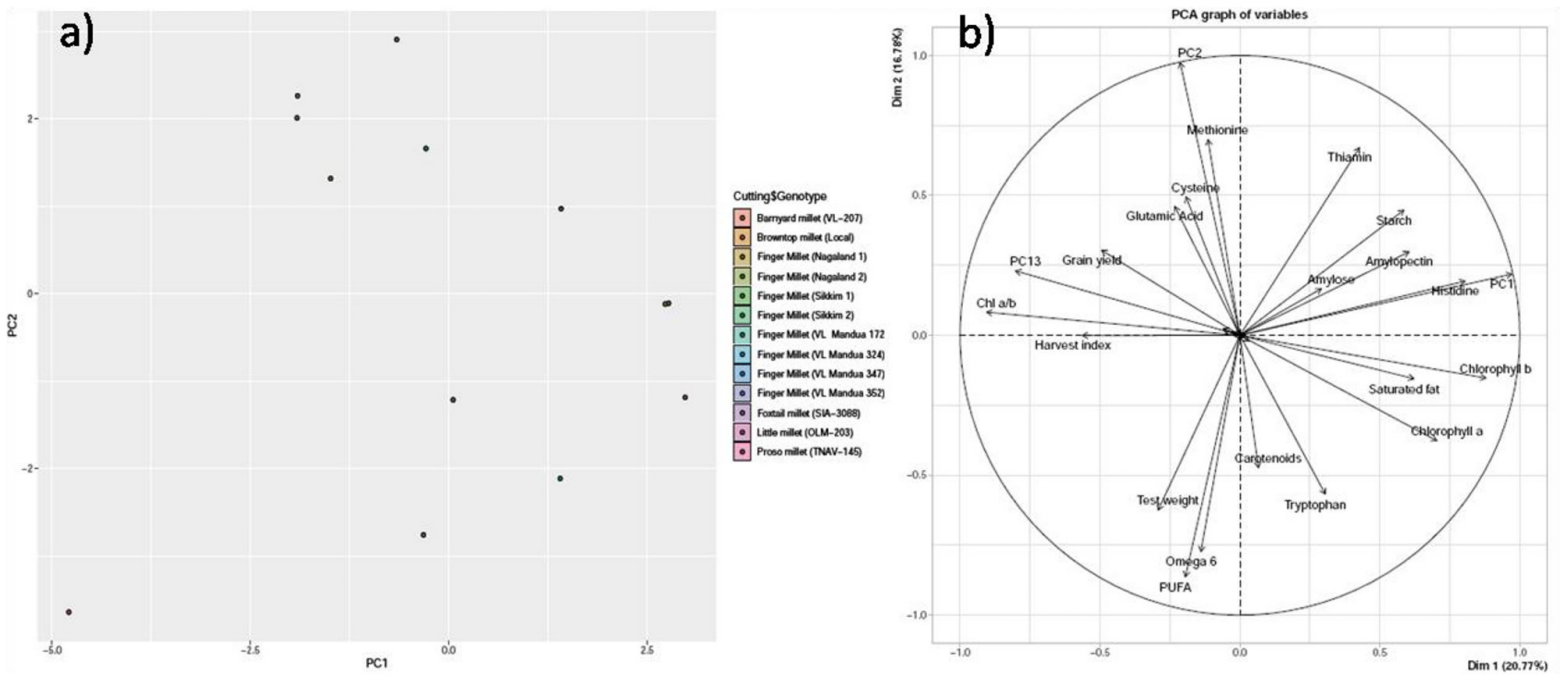
Principal component analysis (PCA) of biochemical parameters. **(a)** Scatter plot and **(b)** Bi-plot. The PCA achieved is not rotated such that each succesfull axis captured as much variance as possible.

PCA revealed that the first three principal components (PC1, PC2, and PC3) explained 66% of the total variation. PC1 accounted for 26% of the total variation, whereas both PC2 and PC3 accounted for 22% of the total variation, respectively (Table 6). Within PC1, chlorophyll a, chlorophyll b, amylose, amylopectin, starch, saturated fat, histidine, and thiamine showed positive loadings, while the rest of the parameters showed negative loadings (Figure 5 and Table 5). In PC2, Chl a/b, grain yield, glutamic acid, cysteine, and thiamine showed positive loading, and the rest of the variables revealed negative loadings (Figure 5 and Table 5). The factor map (component plot) and clustering of all the variables (Figure 5), which reveal the distance between the variables and the origin and assessed the quality of the variables, are both depicted. Positively associated variables are clustered together, while variables with negative correlations are placed on the opposing sides of the plot's origin.

**Table 6:** Factor loadings of biochemical parameters along with the percentage of variance and cumulative variance accounted for each component.

**Parameters**	**PC1**	**PC2**	**PC3**
Chlorophyll a	0.27	−0.25	0.07
Chlorophyll b	0.36	−0.16	0.12
Carotenoids	−0.02	−0.21	0.38
Chl a/b	−0.37	0.12	−0.17
Grain yield	−0.19	0.20	0.28
Harvest index	−0.25	0.071	0.21
Test weight	−0.18	−0.26	−0.07
Amylose	0.13	0.05	0.10
Amylopectin	0.30	0.06	−0.19
Starch	0.30	0.14	−0.09
Omega 6	−0.13	−0.34	0.06
Saturated fat	0.23	−0.12	0.30
PUFA	−0.16	−0.38	0.05
Glutamic acid	−0.06	0.25	0.42
Cysteine	−0.03	0.25	0.31
Histidine	0.35	0.01	0.23
Thiamine	0.24	0.27	−0.09
Methionine	0.01	0.34	−0.27
Tryptophan	0.07	−0.30	−0.28
Standard deviation	2.25	2.07	1.82
Proportion of variance	0.26	0.22	0.17
Cumulative proportion	0.26	0.49	0.66

PUFA, Polyunsaturated fatty acids.

## 4. Discussion

### 4.1. Physiological attributes

There was a need to study the adaptation mechanism of millets in harsh stress environments for their optimum performance and tolerance to stresses (48). The physiological attributes of a plant always play a major role in determining the yield potential and nutritional content in a particular climate and stress condition (49). The root morphological characteristics of millets, such as average root diameter, total root length, and root surface area, play a very important role in essential nutrient uptake from soil and transporting them to the shoots for further help in photosynthesis (48, 50). Among the different millets evaluated in the NEHR of India under organic conditions, the local landrace of finger millet, *Nagaland-1*, recorded the highest average root diameter, root surface area, and root volume, which were statistically at par with landraces *Sikkim-1, Sikkim-2*, and cv. VL Mandua 352. The higher root length, root volume, and surface area of the millet germplasms, VL Mandua 352, *Nagaland-2, Sikkim-1*, etc. determined the overall distribution and functioning of roots and promoted water and nutrient acquisition from the soil, thereby enhancing the crop productivity. There is a need to study the root system architecture of landraces in comparison with high-yielding varieties for improved adaptation to a particular ecosystem with minimum input application (51). This will also help plant breeders to develop suitable varieties by mixing the traits of high-yielding varieties with local landraces for various stress situations (52).

Among different millets evaluated under the organic production system, significantly higher Chl*a*, Chl*b*, and *Chla/b* ratios and carotenoids were recorded for foxtail millet cv. SiA 3088 and finger millet landraces, *Sikkim-2, Sikkim-1*, and *Nagaland-2*, respectively (Table 3). These may be due to the enhancement of photosynthetic efficiency of the millet germplasms as reflected by the improvement of concentration of carotenoids, Chl*a*, and Chl*b* in millet leaves (49). The enhancement of Chl*a*, Chl*b*, and carotenoids is also known to stimulate chlorophyll biosynthesis and subsequently delay the process of senescence of leaves, thereby prolonging the photosynthetic period of plants (53). Table 3 also depicts the stomatal conductance and leaf temperature of different millets grown under organic farming practices. Chatterjee et al. (54) reported that speciation resulted in a steady increase in stomatal conductance (anatomical, *gmax*) in different *Oryza* species. This reduces water loss by transpiration and indicates a good physiological capacity for stomatal regulation (55, 56). Leaf temperature under drought stress conditions has been recognized as an indicator of plant water status (57, 58). The highest stomatal frequency was recorded in finger millet landrace, *Sikkim-2* (471cm^−2^), and little millet cv. OLM 203 observed the highest stomatal size with a value of 1338.7 μm^2^ (Figure 3).

### 4.2. Yield of millets

Among the different millets, finger millet germplasms, *viz*., VL Mandua 352, *Nagaland-2, Sikkim-1*, and foxtail millet cv. SiA 3088 recorded a significantly higher grain yield compared to the rest of the germplasms (Table 3). Better root architecture, higher photosynthetic attributes, and uptake of nutrients and water may have paved for higher photosynthesis (59, 60). The increase in grain yield might be due to the increased photosynthetic activity, which resulted in a higher accumulation of photosynthates and their translocation to sink due to better source and sink channel (61, 62). The increase in grain yield with increased nutrient supply could be explained on the basis of their beneficial effects on yield-attributing characteristics (63). The increase in yield may be due to genetic and environmental factors (64). Pareek and Shaktawat (65) in a study on pearl millet and Munirathnam et al. (66) in a study on foxtail millet have also reported similar findings. Higher HI (Table 3) in selected millet germplasms might be due to dry matter partitioning along with an increased level of nitrogen as reported by Reddy et al. (67).

### 4.3. Nutritional quality parameters

The quality attributes of the millets in this study, as shown in Table 4 and Figure 5, varied across the millets and their germplasms. Generally, millets are reported to contain high concentrations of minerals, essential amino acids, antioxidants, and vitamins, which keep them nutritionally superior compared to other cereals such as rice, wheat, and maize (51, 68). The root is a very sensitive part of plants (here in millets) and responsible for the uptake of water and macro- and micronutrients from the soil (69, 70). Millet lines have better root architectural design (higher root length, root surface area, and diameter, etc.), have better chances to survive under stress conditions, such as flooding, drought, and deficiency of nutrients, and produce nutritionally superior grains compared to others (51). This was proved by nutritionally superior grains with high values of protein, amylose, amylopectin, and starch content of local landraces of finger millets compared to HYVs. Foxtail millet and proso millet are known to contain a relatively higher amount of protein compared to other millets and non-millet cereals (71). Apart from that, the local landraces of finger millets also recorded high amounts of essential amino acids and fatty acids. Lipids are an important source of essential fatty acids, and local landraces of finger millet, foxtail millet, and barnyard millet were found to be excellent sources of omega-3 and polyunsaturated fatty acids (PUFA). Similar findings for essential amino acids in millets were also reported by Amadou et al. (6). The local landraces of finger millet showed significant amounts of essential nutrients compared to other varieties. The experiment also revealed interesting data on the concentrations of essential amino acids, such as histidine, tryptophan, lysine, and methionine. Most of these essential amino acids were found to be significantly higher in local finger millet landraces such as *Sikkim-1* and *Nagaland-2* compared to their corresponding HYVs like VL Mandua 352 or VL Mandua 324. Better uptake of nutrients and higher translocation of photosynthates from source to sink lead to higher protein content in seeds and also higher accumulation of carbohydrates for the local landraces of finger millets (64, 72). These observations corroborate those made by Sharer et al. (73), Chauhan et al. (74), and Nandini and Sridhara (75).

### 4.4. Principal component analysis

Understanding the link between variables can be aided by multivariate statistical analyses such as PCA. These could be useful in clarifying the nature of defining attributes and simplifying the data collection process. The PCA confirmed our findings (Table 5 and Figures 5a, b), with strong and positive correlations among chlorophyll a, chlorophyll b, amylose, amylopectin, starch, saturated fat, histidine, and thiamine in PC 1, while strong and positive correlations were found among Chl a/b, grain yield, glutamic acid, cysteine, and thiamine in PC 2. While the HYVs have high yield potential, they are inferior in nutritional content compared to local millet landraces. In addition, local landraces are adapted to the NEHR, India, and are comparatively more resistant to drought and heavy rainfall than HYVs. This indicates the possibilities for using the local landraces in breeding programs for the production of high-yielding and nutrient-rich varieties.

## 5. Conclusion

The northeastern part of India is organic by default, creating a huge scope for organic millet production. From the above results, it can be concluded that millets can be an important alternative to diversify low-productive mixed farming and supply nutritious food to the people of the northeastern region of India. Apart from nutritional benefits, millets are remarkable crops due to their ability to survive under marginal soil conditions, especially in sloppy and shifting cultivated areas of the NEHR. These features of resilience for the climate-smart crop ensure stable production, enabling local farmers to cultivate millets, which can be a great alternative to rice cultivation in the NEHR. Among the different minor millets studied and evaluated, the foxtail millet cv. SiA 3088, little millet cv. OLM 203, and finger millet germplasms performed well in terms of yield. Along with the HYVs of finger millet, VL Mandua 352 and 347, local landraces of the NEHR, *Nagaland-2* and *Sikkim-1*, showed significantly higher yield and even performed better in biochemical and root traits. The local landraces of finger millets, *viz., Nagaland-2* and *Sikkim-1*, were also found to have superior nutritional quality compared to the HYVs. Therefore, apart from HYVs of finger millets, *viz*., VL Mandua 352 and VL Mandua 347, local landraces such as *Nagaland-2* and *Sikkim-1* should be encouraged to grow among the tribal and hill farmers of the NEHR of India under organic conditions for higher yield and nutritional quality.

## 6. Policy implications

Recent studies have proven that regular consumption of millets along with rice/wheat/maize help in better digestion, nutrition, and reduction in various diseases such as diabetes, arthritis, and heart disease. To popularize millets in the Indian NEHR and to increase its production, several NGOs and local organizations such as the North East Slow Food & Agrobiodiversity Society (NESFAS) and the North Eastern Council (NEC) of the Government of India are constantly working. To remove malnutrition among children and women, our government should give importance to millets in the Midday Meal Scheme, Integrated Child Development Scheme (ICDS), and Public Distribution System (PDS). For enhancing the production as well as consumption of millets along with creating awareness among the masses for food and nutritional security, the United Nations has declared the year 2023 as the International Year of Millets. Research is also needed for using local landraces of millets in breeding programs to produce low-nutrient-demanding and climate-specific millet varieties for food and nutritional security across different ecosystems.

## Data availability statement

The original contributions presented in the study are included in the article/Supplementary material, further inquiries can be directed to the corresponding authors.

## Author contributions

JL: conceptualization, methodology, investigation, monitoring, data curation, and writing of original and final draft. KR: data curation, review, writing, and editing. AD: monitoring, data curation, review, and editing. MA: data analysis, writing of original and final draft, review, and editing. SC, NRaj, SP, AK, SD, SB, MT, and NS: data analysis, review, and editing. VM, NRav, SK, and SH: review, editing, and project administration. All authors contributed to the article and approved the submitted version.
