# Supplementary figures and images for: Evaluation of millets for physio-chemical and root morphological traits suitable for resilient farming and nutritional security in Eastern Himalayas
Source: Front Nutr. 2023 Jul 4;10:1198023. doi: 10.3389/fnut.2023.1198023 (PMC10353539; doi:10.3389/fnut.2023.1198023)

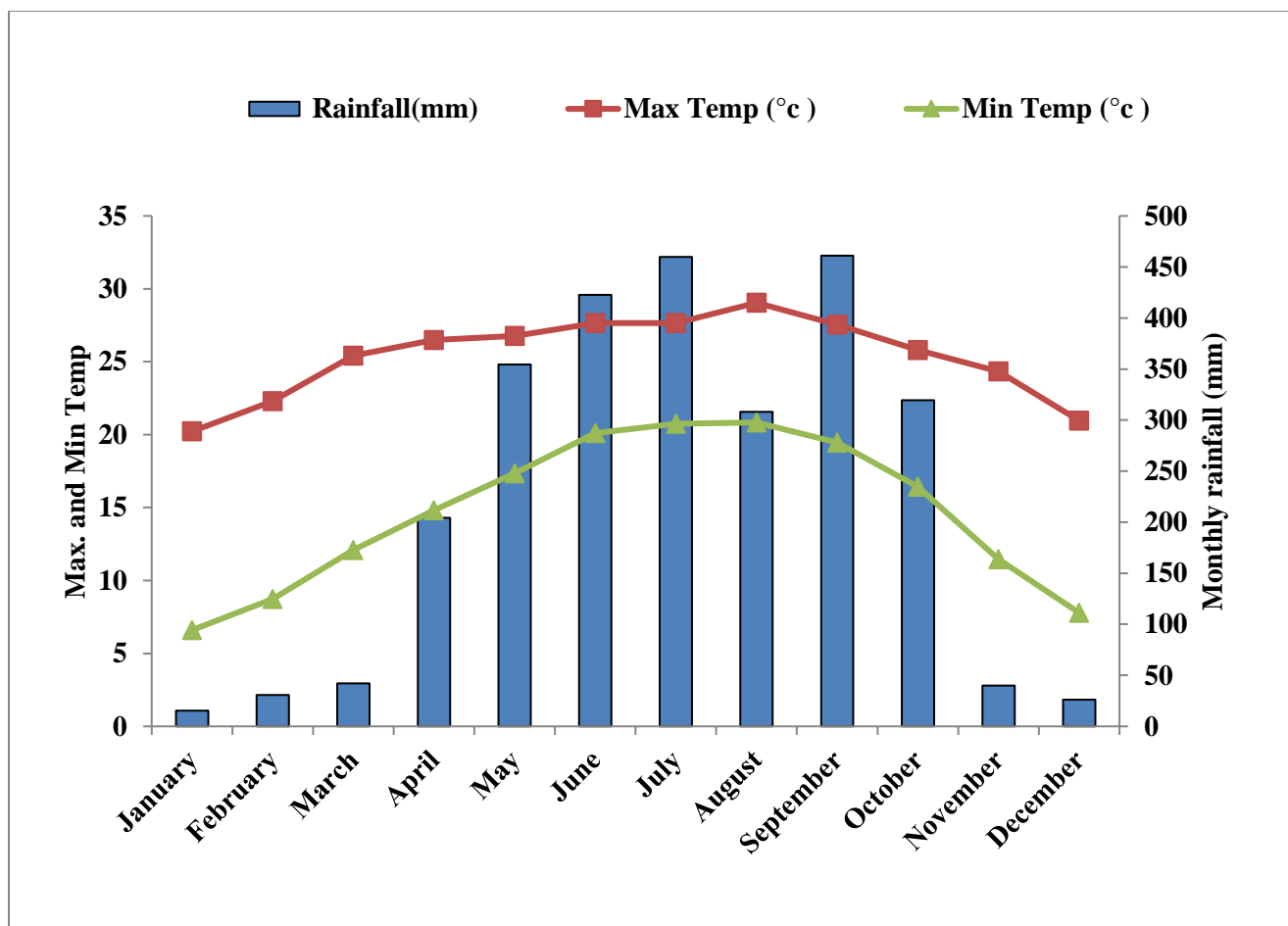

Supp. Figure 1. Monthly data on weather parameters averaged over three years (2018-2020)

Supplement: Supplementary file 1 [file Data_Sheet_1.PDF]
