# Supplementary material for: Evaluation of millets for physio-chemical and root morphological traits suitable for resilient farming and nutritional security in Eastern Himalayas
Source: Front Nutr. 2023 Jul 4;10:1198023. doi: 10.3389/fnut.2023.1198023 (PMC10353539; doi:10.3389/fnut.2023.1198023)
Supplement: Supplementary file 2 [file Table_1.DOCX]

**Supplementary Table 1. Soil properties of the experimental site before initiating the experiment (Year 2018)**

| Soil type | Soil pH | Bulk density (Mg m^-3^) | Soil organic carbon  (g kg^-1^) | Water holding capacity  (%) | Available nitrogen  (kg N ha^-1^) | Available phosphorous (kg P ha^-1^) | Available potassium (kg K ha^-1^) |
| --- | --- | --- | --- | --- | --- | --- | --- |
| Typic Paleudalf | 4.98 | 1.24 | 15.8 | 41.5 | 271.2 | 12.5 | 227.5 |
